# Decoding the spatiotemporal heterogeneity of tumor-associated macrophages

**DOI:** 10.1186/s12943-024-02064-1

**Published:** 2024-07-27

**Authors:** Xiangyuan Chu, Yu Tian, Chao Lv

**Affiliations:** Department of General Surgery, Shengjing Hospital of China Medical University, Shenyang, Liaoning Province 110004 P. R. China

**Keywords:** Tumor-associated macrophages (TAMs), Tumor microenvironment (TME), Cancer immunotherapy, Spatial multiomics, Drug resistance

## Abstract

Tumor-associated macrophages (TAMs) are pivotal in cancer progression, influencing tumor growth, angiogenesis, and immune evasion. This review explores the spatial and temporal heterogeneity of TAMs within the tumor microenvironment (TME), highlighting their diverse subtypes, origins, and functions. Advanced technologies such as single-cell sequencing and spatial multi-omics have elucidated the intricate interactions between TAMs and other TME components, revealing the mechanisms behind their recruitment, polarization, and distribution. Key findings demonstrate that TAMs support tumor vascularization, promote epithelial-mesenchymal transition (EMT), and modulate extracellular matrix (ECM) remodeling, etc., thereby enhancing tumor invasiveness and metastasis. Understanding these complex dynamics offers new therapeutic targets for disrupting TAM-mediated pathways and overcoming drug resistance. This review underscores the potential of targeting TAMs to develop innovative cancer therapies, emphasizing the need for further research into their spatial characteristics and functional roles within the TME.

## Introduction

In current research, understanding the dynamics of tumor-associated macrophages (TAMs) within the intricate context of the tumor microenvironment (TME) has become paramount. The TME constitutes the immediate surroundings where tumors thrive, encompassing blood vessels, stromal cells, immune cells, and a milieu of signaling molecules, all pivotal in tumor progression. Macrophages, key immune cells tasked with phagocytosis and pathogen clearance, are pivotal players within the TME [1]. Within this context, TAMs, shaped by diverse microenvironmental cues, can adopt either tumor-promoting (M2-type) or tumor-suppressing (M1-type) phenotypes [2], making them a prime target for therapeutic intervention [3].

Studies indicate that TAMs predominantly support tumor growth and invasiveness, orchestrating a complex interplay with various TME constituents to foster tumor immune evasion. This recognition has underscored macrophages as promising therapeutic targets. Recent technological advancements, notably single-cell sequencing and spatial multi-omics technologies, have unveiled the spatial specificity and temporal heterogeneity of TAMs in the TME. TAMs exhibit distinct subtypes and functions at different tumor stages and locations, posing challenges to effective therapeutic strategies [4].

Within the TME, TAMs are strategically distributed around blood vessels and tumor cells, interacting with various cells, including stromal and immune cells [5, 6]. This diversity is shaped by numerous factors, such as signaling molecules secreted by tumor and stromal cells. TAMs primarily originate from two sources: tissue-resident macrophages (TRMs) and monocyte-derived macrophages (MDMs), with their distinct distributions influenced by chemokines, inflammatory factors, and other TME elements [7, 8].

TAMs engage in multifaceted interactions within the TME: (1) Recruitment by vascular endothelial and perivascular cells, leading to polarization and the expression of angiogenic factors that promote tumor angiogenesis [9, 10]. (2) Conversion to the M2 phenotype by tumor-derived inflammatory mediators, which in turn promote tumor invasion [11, 12]. (3) Co-localization with tumor cells undergoing epithelial-mesenchymal transition (EMT), facilitating tumor immune escape [13]. (4) Recruitment by cancer-associated fibroblasts (CAFs), and recruited TAMs promote the role of CAFs in promoting extracellular matrix remodeling and facilitating tumor invasion [14, 15]. (5) Recruitment and “education” by tumor cells via signaling molecules such as extracellular vesicles (EVs), cytokines, and chemokines, transforming them into pro-tumorigenic agents [16, 17]. (6) Interaction with other immune cells (T cells, B cells, NK cells, MDSCs) in the TME, which suppresses T cell infiltration into tumor tissues and promotes tumor growth [18]. In conclusion, the dynamics of TAMs evolves throughout tumor development, regulated by multiple signaling molecules in the TME.

Due to the fact that TAMs form a complex “network” of interactions in the TME and play an important role in tumor progression, targeting the relevant molecules may cause a series of events that could dramatically change the approach to cancer treatment. This approach has great potential as TAMs have a wide range of roles in tumors. This review mainly focuses on the spatiotemporal distribution characteristics of TAMs and the mechanisms behind them. We first introduce the different subtypes, functions, and distribution characteristics of TAMs. We then explore the reasons for their spatiotemporal heterogeneity, considering their co-localization and interactions with other cells, their migration, and dynamic evolution during tumor progression. We examine their origins, involvement in vascularization, inflammation, EMT, extracellular matrix remodeling, and interactions with immune cells. Additionally, we summarize current research methods and strategies for addressing TAMs’ spatial distribution and heterogeneity. Finally, we review current therapies targeting TAMs and propose new strategies, believing that these approaches can lead to innovative tumor therapies and overcome macrophage-mediated drug resistance.

## Spatiotemporal heterogeneity of TAMs

TAMs variate not only across spatial dimensions within the TME but also over time. These variations in TAM characteristics, functions, and distribution significantly impact the prognosis of tumors. The advent of single-cell sequencing and spatial multi-omics has revolutionized our understanding of TAMs. These technologies have uncovered that different TAM subtypes exhibit varied distributions and interactions with other cells within the TME, which can lead to drug resistance and therapeutic interference. The use of these typing methods, though beneficial, categorizes macrophages predominantly into two extremes based on phenotypic characteristics determined by cytokine profiles and surface markers. This often complicates the complete differentiation of TAMs [19, 20]. Spatial transcriptomics and immunohistochemistry have further exposed the diversity within TAMs in tumors, revealing instances of M1 and M2 co-expression [21]. Therefore, new typing methods are strongly needed to better characterize macrophage diversity.

### Characterization and diversity of TAM subtypes

**TAM Subtypes basing on surface marker or gene expression** Most current studies primarily classify TAM subtypes based on differences in surface marker expression, functionalities, genes, and proteins. For instance, in gastric cancer, an analysis of tissues from 56 human cases through multiplex immunohistochemistry (mIHC) identified seven distinct TAM populations based on CD68, CD206, and CD163 expressions, uncovering that not all M2 macrophages directly influence tumor progression [22]. Another study identified three TAM subtypes in the microenvironment of Granulomatous slack skin, exhibiting different functionalities where CD206(+)/CD163(+) showed an M2-like phenotype, potentially interacting with T cells to promote tumor progression [23].

These studies primarily classify macrophages based on differences in surface markers. However, other research takes a different approach by focusing on gene expression. A study on breast cancer identified two TAM subtypes differentiated by the expression of three specific genes, revealing distinct locations and tumor-supporting functions, thus contributing to the heterogeneity within TAMs [24].

**Regional Variability in TAM Functions** This diversity in TAM subtypes and functions across different tumor regions is critical for understanding the tumor ecosystem, particularly at the tumor margins where cell infiltration and invasion occur. This understanding is vital for exploring tumor metastasis mechanisms, predicting tumor progression, and developing effective new therapies. One notable study utilized a novel imaging technique with nanoscale resolution (Stereo-seq) to show that TAMs are not uniformly distributed within tumor tissues but instead have a structured spatial distribution, predominantly as M2 macrophages in the invasive zones of the tumor margins, which correlates with a higher recurrence rate [25]. Further studies on gastric cancer showed that different TAM subtypes expressed at the tumor core versus the margins influence tumor dynamics significantly, while more M1 subtype in the tumor core and more M2 subtype in the margins [22]. In the invasive fronts, macrophages expressed histones, pro-matrix metalloprotein9,IL-10, CD100, PD-L1+, EGF, CD80 + and CD86 + to promote immunosuppression and promote the invasion of cancer cells [26]. In addition, in an analysis of single-cell sequencing and IHC in colorectal cancer, it was shown that the CD163+/CD68 + ratio was significantly higher in the tumor margin than in the tumor core, and that this ratio was positively correlated with Lymphatic invasion, tumor invasion, and TNM classification [27].

Tissue-resident macrophages (TRMs) and MDMs display distinct patterns of aggregation within tumors and exhibit dynamic changes in their populations as the tumor progresses. TRMs are categorized into various subsets based on specific functions and locations, including Kupffer cells, erythromedullary macrophages, osteoclasts, alveolar macrophages, and microglial cells in the liver, spleen, bone, lungs, and brain, respectively [28]. In glioblastoma research, it has been observed that MDMs primarily accumulate in the tumor’s core, whereas microglia-derived macrophages are more frequently found surrounding the tumor [7, 29]. Further studies on small cell lung carcinoma reveal that as the tumor advances, there is a noticeable decrease in TRMs and an increase in MDMs. During the process of tumor invasion, tumor cells initially cluster around TRMs, which gradually migrate toward the periphery of the tumor as the invasion progresses [30].

These results may suggest that macrophages in different parts of the tumor differentiate into different phenotypes thereby influencing tumor progression and invasion. This spatial heterogeneity may be due to the interaction of each ecological niche and TAMs in the tumor tissue, which may include angiogenesis, metabolism, and tumor stroma.

**Heterogeneity and Vascular Interactions** mIHC and spatial transcriptomic analyses of lung adenocarcinoma tissues have identified several macrophage subtypes, including CD68+, CD68 + D163+, CD68 + CD206+. Notably, the CD68 + CD206 + subtype was predominantly found in vascularized regions [31]. In these areas, macrophages expressing TIE2^Hi^MRC^Hi^ CD163^Hi^TLR4^Hi^ surface markers are known to stimulate tumor migration, angiogenesis, and immunosuppression [26]. Additionally, TAMs in the perivascular regions predominantly express MRC1, while those in hypoxic regions express ARG1 [32].

These findings suggest a spatially-dependent interaction between TAMs and the vasculature, where TAMs may promote angiogenesis, and the vasculature may further tumor progression by recruiting macrophages that enhance angiogenic processes. Further details of this interaction will be explored in the subsequent section. A similar pattern has been observed in the study of brain gliomas, where IHC and genetic analysis have shown variations in the spatial distribution and functional expression of different macrophage sources. Specifically, microglioma cell-derived macrophages are localized in the peritumor region, whereas bone marrow-derived macrophages, identified as Mo-TAM_inf. clusters (IBA1+/CXCL3+), are primarily found in the perinecrotic region and aggregate at the borders, predominantly in the perivascular area, suggesting a role in promoting angiogenesis [33].

Furthermore, IL1β + TAMs, a subset of macrophages in pancreatic ductal adenocarcinoma, have been identified and analyzed for their transcriptomic profile, which revealed their primary functions in promoting inflammatory responses, leukocyte recruitment, and angiogenic effects. These macrophages are correlated with poor prognosis and are enriched in hypoxic regions [34].

**Dynamic Spatial and Temporal Patterns** TAMs are dynamic and exhibit temporal changes in their spatial distribution. The temporal heterogeneity of TAMs is mainly manifested by changes in the subtypes, functions, and locations of TAMs at different periods and stages of tumor development. In the early stage of tumor, TAMs mainly showed M1 subtype; while with tumor invasion, metastasis, M2 subtype dominated. In B6-WT hosts, the distribution of CD68 + TAMs is initially uniform but becomes regionally clustered by 3–4 weeks. In contrast, in B6-SCID hosts, CD68 + TAMs remain uniformly distributed throughout the progression of glioblastoma multiforme, demonstrating a highly dynamic spatial pattern that differs significantly from that in B6-WT hosts. Both early and later stages show clustering in distinct regions [29]. A study on colorectal cancer revealed significant differences in macrophage behavior between early-stage and advanced/metastatic tumors. Macrophages from early-stage tumors exhibited an elevated expression of pro-inflammatory and chemokine factors. This trend suggests that macrophages in the early stages of tumor development are more inflammatory, while those in advanced stages display higher levels of anti-inflammatory markers. This temporal pattern of inflammatory expression indicates a shift in macrophage function as the tumor progresses [35]. Additionally, in metastatic tumors, MRC1 + CCL18 + macrophages, SPP1 + macrophages, and neutrophils were found to have significantly enhanced expression, suggesting a correlation between macrophage phenotype and tumor stage [36].

Understanding the temporal heterogeneity of TAMs is critical for devising more effective cancer treatment strategies, as it allows for the targeting of TAMs at specific stages of tumor development. However, current studies on this aspect face significant limitations due to the dynamic nature of TAMs. This dynamism requires frequent sampling over various stages, substantially increasing experimental complexity. Meanwhile, the phenotype and function of TAMs evolve with time and TME, capturing these changes dynamically and continuously presents substantial clinical and ethical challenges [37].

TAMs exhibit considerable heterogeneity, complicating the definition and tracking of specific subgroups. Understanding the dynamic and diverse nature of TAMs is crucial for developing targeted therapeutic strategies and improving cancer treatment outcomes. The spatiotemporal heterogeneity of TAMs is associated with varying tumor prognoses. Studies have shown that the number of M2 macrophages and their distribution within the tumor correlate with patient prognosis and survival. For instance, in lung cancer, the density and spatial distribution of TAMs were linked to overall survival, with closer proximity of tumor cells to TAMs associated with tumor cell survival, whereas hypoxia correlated with the accumulation of M2 TAMs. Lower density of M1 TAMs and higher proximity to M2 TAMs were associated with reduced survival [38]. This suggests that TAMs may be tumor-specific, and in different TME, TAMs are stimulated differently, express different proteins, secrete characteristic cytokines and chemokines, and ultimately have different effects on tumors. A study showed that IL4I1 + macrophages were associated with tumor cell phagocytosis and predicted a positive prognosis for colon cancer patients, whereas SPP1 + macrophages were associated with hypoxia and necrotic tumor areas and predicted a poor prognosis [39]. Therefore, the heterogeneity of TAMs, both spatially and temporally, highlights the complexity of the tumor ecosystem and necessitates a deeper understanding to harness this knowledge for therapeutic advances.

## Causes of TAMs heterogeneity

**Fig. 1:**
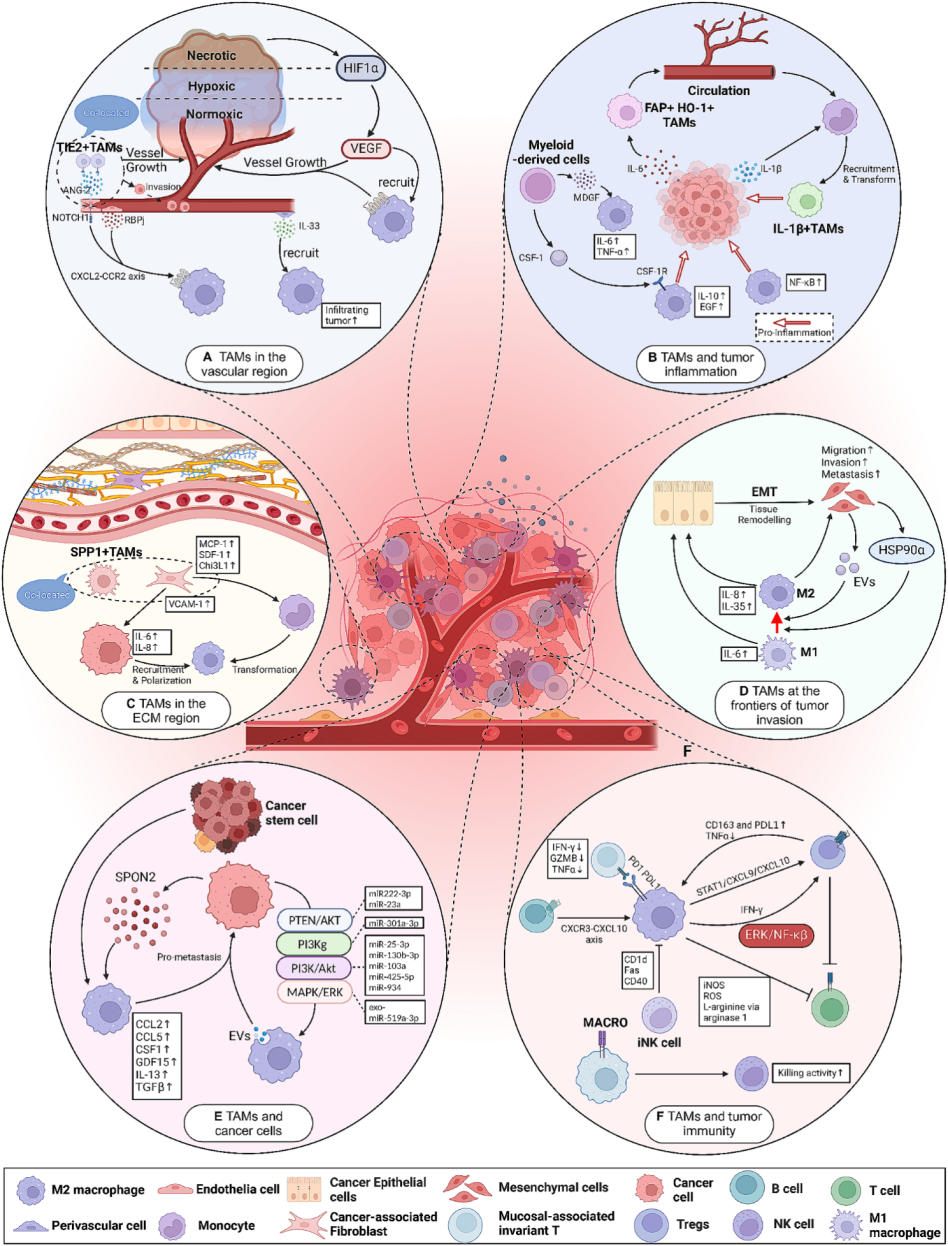
The factors influencing the spatiotemporal heterogeneity of tumor-associated macrophages **(A)** TAMs in vascular regions. TAMs can be recruited directly or indirectly to specific regions within the tumor by tumor vessels, vascular endothelial cells, pericytes, and others. Tumor hypoxia releases HIF1α, which induces macrophages to promote tumor angiogenesis through VEGF. Vascular endothelial cells secrete ANG2 can recruit TIE2 + TAMs through the TIE2 + pathway, which increases vascular permeability and promotes tumor cell endocytosis. ECs can also promote macrophage recruitment and polarization through the Notch signaling/CXCL2 or RBPJ/CXCL2 axis. Perivascular cells can secrete IL-33 to promote macrophage recruitment. **(B)** Inflammatory microenvironment in TAMs and TME. Various tumor-derived mediators (CSF-1, CCL2, VEGF-A, TNF-a, etc.) can induce macrophage chemotaxis into tumor tissues and promote their release of inflammatory cytokines IL-6 and TGF-α, which promote tumor growth. Tumor cells recruit monocytes to the tumor via IL-1β and polarize them to IL-1β + TAMs. CSF1, MDGF facilitate the recruitment of macrophages to the tumor and polarize them to an M2-like phenotype. These recruited macrophages express pro-tumorigenic effects. In addition, IL-6 secretion by tumor cells promotes the entry of FAP + HO-1 + TAMs into the vasculature. **(C)** TAMs in the ECM region. CAFs can promote ECM remodeling by secreting various factors (MCP-1, SDF-1, Chi3L1, etc.) to promote monocyte recruitment and differentiation into M2-like macrophages. The remodeled ECM inhibits immune cell infiltration and facilitates tumor invasion and metastasis. **(D)** TAMs and the EMT process. Cells undergoing epithelial mesenchymal transition can directly secrete HSP90α, which induces macrophage M2 polarization. EMT-CRC cells can also promote M2-like polarization of macrophages by directly transferring exosomes to macrophages. TAMs can also secrete IL-6, IL-8, and IL-35 to induce EMT in cancer cells. **(E)** TAMs and tumor cells. Tumor cells can secrete RNA via EV through various signaling pathways (miR222-3p miR-23a/PTEN/AKT, miR-301a-3p/PI3Kg, miR-25-3p, miR-130b-3p, miR-103a, miR-425-5p, miR-934/PI3K/Akt, exo-miR-519a 3p/MAPK/ERK), promoting M2 macrophage recruitment. In addition, tumor cells can also secrete relevant mediators to promote the recruitment of TAMs. **(F)** TAMs and other immune cells. M2 can inhibit T cell infiltration through CXCL10-CXCR3 and Tregs interactions. Tregs promote macrophage expression of M2 markers (CD163, PDL1, etc.).TAMs can also directly inhibit the proliferation of CD8 + T cells by secreting various factors. TAMs can recruit B cells through the CXCR3- TAMs can recruit B cells through the CXCR3-CXCL10 axis. TAMs can also interact with MAIT through the PD1-PDL1 pathway. In addition, expression of MACRO + TAMs can also enhance NK cell function. iNK cells can also inhibit M2 macrophages through molecules such as CD1d, Fas, and CD40

The niche of the original tumor is affected by a variety of stem cells and immune cells. In the initial stages of tumorigenesis, cancer cells may be a target for elimination by the immune system. Fibroblasts and macrophages may initially serve to inhibit tumor growth, but as the cancer progresses, these cells may develop pro-tumorigenic functions. For example, TAMs may support both angiogenesis and invasion by producing growth factors, cytokines, and proteinases. CAFs can be induced to produce ECM protein and vasculogenic factors, such as vascular endothelial growth factor-A, thus complicating the intricate interactions within primary TME. During intravascular infiltration, macrophages are localized in the perivascular microenvironment and assist cancer cells in crossing the vascular barrier - a critical step in the formation of metastatic TME.(Fig. 1).

### TAMs biology: discussing the different origins or recruitment mechanisms that give rise to diverse subsets of TAMs

It is widely recognized that TAMs originate from two primary sources: TRMs and MDMs. TRMs typically develop from embryonic yolk sac cells, while MDMs are primarily derived from bone marrow [40, 41]. TRMs are found in most organs, where they differentiate into organ-specific macrophages during embryonic development, such as Kupffer cells in the liver, erythrocytic myeloid macrophages in the spleen, alveolar macrophages in the lungs, and microglia in the brain [2].

These macrophages, from differing origins, assume unique locations and roles within the TME. For example, in different cancers such as non-small cell lung cancer, breast cancer and glioma, distinctions are observed in the positioning of pre-existing TRMs and MDMs. In glioblastoma, MDMs are predominantly found in the tumor core, whereas microglia-derived TAMs are more common at the tumor periphery [7, 8]. Similarly, in lung cancer and glioma, embryonic-derived TRMs typically localize at the tumor periphery, while MDMs infiltrate the tumor core [5], may be influenced by the specific signaling molecules encountered in different TME.

In the MN-MCA1 mouse cancer model, monocyte-derived HO-1 + TAMs have also been demonstrated to preferentially localize to the infiltrative margins of primary tumors and metastatic foci through a mechanism dependent on, and coordinated with, Nrf2 activation and to play a key role in metastatic TME that promotes angiogenesis and EMT [42]. Typically, embryonic-derived TRMs exhibit an anti-inflammatory phenotype and are implicated in post-traumatic tissue repair, whereas bone marrow-derived macrophages infiltrating post-traumatic tissues exhibit a pro-inflammatory phenotype [43]. In a mouse lung model, TAMs were identified as a mix of tissue-resident mesenchymal macrophages and CCR2-dependent recruited macrophages (MDMs), each group showing distinct functions and distribution patterns. Tissue-resident mesenchymal macrophages primarily support tumor cell growth in vivo, while MDMs accumulation correlates with increased tumor spread [44]. Persistent TRMs in pancreatic ductal adenocarcinoma predominantly accumulate in the ECM, playing a key role in fibrosis and tumor progression [45]. In terms of cytokine expression, MDMs primarily express IL-10, whereas Arg-1 levels are significantly higher in microglia [46].

**Causes of TAM Variability** The diversity in TAM characteristics stems from various factors, including exposure to stimulatory signals and interactions with tumor cells, stromal cells, and other immune components. Initially, TRMs are the first macrophages contacted during tumorigenesis, followed by various metabolic activities that enable the recruitment of peripheral monocytes [19]. The recruitment and retention of bone marrow-derived monocytes at metastatic sites are largely governed by the CCR2-CCL2 axis. TAM-produced CCL4, along with CCL5, may also facilitate the recruitment of bone marrow-derived monocytes to the tumor site [12]. Additionally, VEGFA and Sema 3 A released by tumor cells can activate NRP1, which in turn triggers VEGFR1 activation and enhances TAM recruitment in gliomas [19]. IL-34 from tumor cells mediates monocyte attachment to the vascular endothelial layer through CSF-1R binding on peripheral monocytes [19]. In most cancers, macrophages are enriched by infiltrating MDMs that are recruited from the periphery in response to signals from the TME (e.g., CCL2, CCL9, CSF-1, VEGF, and TGF-b), and their in situ phenotype is shaped by a combination of organ- and tumor-derived signals [2, 47].

### Vascularization: how tumor vasculature influences TAM distribution and behavior

Neovascularization plays a vital role in the progression and metastasis of tumors, where tumor vasculature regulates tumor growth through oxygen supply and supports tumor metastasis through cancer cell spread. In many solid tumors, microvessel density is associated with angiogenesis, metastatic risk, and the prognosis.

TAMs often specifically localize around tumor vasculature, with multiple subtypes identified near these regions. In glioblastoma, for instance, macrophages are more prevalent around blood vessels than in other regions, especially during proliferative microvascularization [19]. A subset of TAMs expressing the angiopoietin receptor Tie-2 accumulate at perivascular sites across various malignancies, facilitating angiogenesis, tumor growth, and tumor recurrence post-chemotherapy [48, 49]. Additionally, LYVE-1 + TAMs, recruited from outside the TME, cluster in perivascular nests mediated by IL-6, with TAM surface CCR5 playing a role in ecological niche coordination [50]. In human hepatocellular carcinoma, CCR2 + TAMs localize within the stroma and align with pathogenic vascularization to promote angiogenesis [51]. The MMTV-PyMT mouse model of spontaneous breast cancer describes a specific subpopulation of Lyve-1 + TAMs around blood vessels, which coordinate the expansion of a pericyte-like mesenchymal stromal cell population, creating pro-angiogenic perivascular ecological niches [52]. These perivascular ecotopes serve as primary sites for peripheral monocyte recruitment, where they differentiate into macrophages expressing pro-angiogenic and pro-tumorigenic markers such as VEGFA, CCR2, and TIE2 [53, 54].

The interaction between TAMs and tumor vasculature is mutual and influential. In B6-WT hosts, tumor vasculature transforms from a dense, regular network to a sparse, congested, and tortuous system. Concurrently, CD68 + TAMs migrate from perivascular locations to vascular-poor regions, generating new tumor vessels and promoting metastasis. This spatial organization mirrors the dilated vascular changes observed in gliomas [29]. Tumor cells and vascular-associated cells recruit and stimulate TAM distribution and differentiation by secreting various cytokines, enhancing tumor angiogenesis and progression.

Moreover, tumor hypoxia significantly influences M2-specific TAM distribution around vasculature. Hypoxia-induced CXCL12 and ANGPT2 expression stimulated the recruitment and revascularization of perivascular aggregates of TAMs expressing CXCR4 and TIE2, respectively. The ANGPT2-TIE2 signaling pathway promotes pro-angiogenic interactions between perivascular TAMs and neoplastic tumor-associated vessels, while the TIE2 + perivascular TAMs-induced VEGF-A signaling pathway leads to a vascular transient increase in permeability that promotes endocytosis by cancer cells [37, 55]. Lactate, a by-product of both aerobic and anaerobic glycolysis from tumor cells, plays a crucial role by inducing VEGF expression and M2-like polarization of TAMs [56].

Vascular endothelial cells (ECs) recruit TAMs through mechanisms like Notch signaling, which promotes MDMs recruitment to the TME mediated by the RBPj/CXCL2 axis [9]. ECs also secrete Ang-2, enhancing VEGF-dependent angiogenesis and promoting CCR2 + MDMs infiltration through increased vascular permeability [57, 58]. Perivascular cells, including fibroblasts, recruit and polarize M2 phenotypic macrophages, with TAMs recruiting pericytes to form pro-angiogenic perivascular niches [59]. Additionally, perivascular cells secrete IL-33, stimulated by PDGF-BB and PDGFRβ, enhancing TAM migration and infiltration via ST2-dependent mechanisms [57, 60].

TAMs directly and indirectly affect tumor vasculature, promoting neovascularization through upregulation of VEGF, PDGF, and TGF-β, and increasing production of angiogenesis-associated growth factors. M2 macrophages correlate positively with microvessel density in pancreatic ductal adenocarcinoma, with M2-derived exosomes enhancing angiogenesis in vitro [61]. The presence of PYCR1 in THCA correlates with SPP1 + angiogenesis-associated macrophages, highlighting their potential pro-tumorigenic role [62]. Enhanced TAM infiltration is linked to increased angiogenesis and tumor progression, with TAMs orchestrating resistance to anti-angiogenic treatments through mechanisms that boost pro-angiogenic factor production (VEGF and FGF) and promote malignant tumor cell behaviors [10]. A unique TAM population is recruited via the Sema3A-Nrp1 pathway to avascular, hypoxic tumor regions, acquiring pro-angiogenic and immunosuppressive properties [12, 63]. Additionally, TAM expression in hypoxic environments upregulates REDD1, inhibiting glycolysis and promoting abnormal vessel formation, leading to metastasis [64]. Compared to TREM2-TAM, TREM2 + TAMs secrete lower levels of CXCL9 but higher levels of galactagogue lectin-1, which promotes PD-L1 overexpression in vascular endothelial cells, impedes CD8 + T cell recruitment, and fosters tumor growth [65].

In summary, tumor neovascularization is a crucial step in tumor invasion and metastasis, and TAMs play a pivotal role in this process. Targeting the interactions between TAMs and tumor vasculature, including the signaling pathways and cytokines involved, represents a promising strategy to inhibit tumor progression and overcome resistance to current therapies.

### Inflammation: the impact of inflammatory signals on TAM polarization and activation states

Inflammation is a crucial driver of tumor development, with macrophages playing a central role in both inflammation and carcinogenesis. In hepatocellular carcinoma, for instance, it has been observed that tumor-initiating cells recruit polarized M2-like macrophages which aid in immune evasion. Various inflammatory factors secreted by tumor cells and other sources contribute to the recruitment and polarization of TAMs through multiple molecular mechanisms, which will be briefly outlined below.

Key mediators derived from tumors that drive macrophage mobilization and activation include CSF-1, G-CSF, GM-CSF, CCL2, CXCL1, CXCL2, IL-1β, IL-6, TNF-α, VEGF-A, and the 3 A proteins [66]. Myeloid-derived growth factor promotes chemotaxis of macrophages into tumor tissues, enhancing their release of inflammatory cytokines such as IL-6 and TNF-α. These cytokines remodel the TME, supporting tumor angiogenesis and metastasis [67]. Mitochondrial reactive oxygen species also significantly impact TAMs; mitochondrial Lon, a chaperone protein, triggers ROS-dependent inflammatory cytokine production, including TGF-β, IL-6, IL-13, and VEGF-A, which activate angiogenesis, and M2 macrophage polarization. Furthermore, activation of Lon expression follows macrophage activation and M2 polarization, enhancing the immunosuppressive microenvironment and metastatic potential of M2 macrophages in the TME [68]. The NF-κB pathway is a critical regulator of inflammation, with its activation in cancer cells leading to cytokine and chemokine secretion that attracts M2 macrophages to the TME, correlating with poor tumor prognosis [69, 70]. Additionally, a monocyte-derived STAB1 + TREM2 high-fat-associated macrophage subpopulation has been identified as immunosuppressive and closely associated with inflammatory cancer-associated fibroblasts (iCAFs). This relationship emphasizes the role of the CXCL12-CXCR4 axis in monocyte recruitment at tumor sites, regulating breast cancer metastasis, cancer cell migration, EMT, and intra-tumor Treg recruitment [71].

IL-33 levels, specifically elevated in human pancreatic ductal adenocarcinoma, correlate positively with tumor inflammation. IL-33-stimulated macrophages are the primary source of CXCL3, which is highly upregulated in pancreatic ductal adenocarcinoma compared to other cancer types. CXCL3 activation of CXCR2 induces a transition from CAF to myofibroblast-like CAFs (myoCAFs) and uniquely upregulates α-smooth muscle actin [72]. IL-1β signaling in tumor cells recruits monocytes, triggering the IL-1β + TAM state that drives inflammatory reprogramming in neighboring pancreatic ductal adenocarcinoma cells, enhancing synthesis of PGE2, TNF, and other factors that perpetuate the IL-1β + TAM state [34].

IL-6 regulates the FAP + HO-1 + macrophage phenotype and is expressed directly by macrophages within tumors. It modulates the potential for HO-1 expression through autocrine and paracrine signaling while stimulating FAP (fibroblast activation protein α) expression in a collagen-rich microenvironment. This activity promotes tumor cell migration across the endothelium and enhances tumor metastasis [73]. Additionally, IL-6 shifts macrophage polarization towards pro-tumorigenic phenotypes, resulting in the production of CC-chemokine-ligand-20 within the colorectal cancer microenvironment, which promotes cancer-associated colitis progression by recruiting B cells expressing CC chemokine receptor 6 [74].

CSF1, a myeloid cytokine released during infection and inflammation, facilitates the recruitment of MDMs to the tumor bed and polarizes them to an M2-like phenotype. This process promotes fatty acid oxidation as well as secretion of pro-tumorigenic and immunosuppressive factors such as EGF and IL-10 [4]. In a gastric cancer model, the simultaneous over-expression of COX-2 and mPGES-1 in epithelial cells leads to the accumulation of TAM, which induces CCL2 secretion through stromal recruitment of PGE2 [75].

In addition to the roles previously described, complement C3 also plays a potential role in TAM recruitment and polarization. In mouse sarcomagenesis models, complement C3 acts upstream in the cascade that leads to macrophage recruitment and their functional localization within tumors, highlighting its critical role in shaping the immune landscape [76].

Moreover, inflammation-recruited TAMs possess specific functions that facilitate tumor growth and metastasis. They form a conducive microenvironment for tumor expansion by secreting various inflammatory mediators. Tumor-infiltrating monocytes, for instance, express elevated levels of inflammatory cytokines and chemokines such as IL-1β, CXCL2 ,CCL4, and CXCR4, along with cell growth regulators like AREG and EGR1, and markers of tissue residency such as NR4A1, NR4A2, and NLRP3. These cells also exhibit upregulated nuclear factor kB (NF-kB) signaling genes NFKB1 and NFKBIA, further contributing to the inflammatory milieu [62].

In the context of pancreatic ductal adenocarcinoma, M2-type macrophages, stimulated by tumor inflammation and Ig-G, release IL-1β, which not only promotes the EMT phenotype but also increases metastatic potential [77]. Additionally, SPP1 + macrophages undergo a functional reprogramming within the TME, exhibiting high expression of genes associated with inflammatory fibrosis such as CTSB and LGALS3, and those involved in lipid metabolism like APOE and APOC1. This reprogramming is induced by CAF-derived ligands including CSCF1, FGF1, PGF, TGFB3, and TIMP1 [78]. Moreover, IL15Rα + TAMs, which express high levels of IL-15Rα, not only reduce CX3C chemokine ligand 1 levels in tumor cells but also inhibit CD8 + T-cell recruitment through the IL-15/IL-15Rα complex, contributing to poorer survival outcomes in breast cancer patients [79]. Additionally, monocytes recruited in inflammatory breast cancer secrete IL-8 and GRO, which drive a feed-forward loop of EMT, demonstrating how TAM-specific recruitment and activity support tumor progression [80].

Together, these insights underline the complex and multifaceted roles TAMs play in promoting tumor progression and shaping the tumor microenvironment through both direct and indirect mechanisms.

### Epithelial to mesenchymal transition (EMT): how cellular transitions within tumors affect TAM phenotypes

EMT is a process that is reversible, in which epithelial cells lose their attachment to the basement membrane and intercellular connections and are transformed into mesenchymal cells with migratory and invasive properties. This transformation involves changes at the cellular, genetic, physiological and metabolic levels, which together promote tumor migration and drug resistance [81].

It has been observed that the EMT process frequently occurs at the forefront of tumor invasion, where TAMs are also commonly found, suggesting a symbiotic relationship where cancer cells undergoing EMT may influence the behavior of nearby macrophages. For example, in gastric cancer, co-localization of gastric cancer mesenchymal stem cells and TAMs, predominantly of the M2 subtype, has been demonstrated by mIHC [82]. M2 macrophage-derived TGFβ1, in particular, have been shown to induce EMT and drug resistance in cholangiocarcinoma cells via the atypical protein kinase C iota-mediated NF-κB signaling pathway [13]. Additionally, spatial proximity between EMT cells and TAMs has been noted in the microenvironment of breast cancer, where mesenchymal-like breast cancer cells activate TAM-like macrophage phenotypes through GMCSF, and vice versa; CCL18 TAMs induce cancer cell EMT, creating a positive feedback loop that correlates with patient survival [83].

Multiple studies have shown that during EMT, tumor cells secrete a series of cytokines, microRNAs, and chemokines that promote TAM recruitment and polarization. EMT-CRC cells can transfer exosomes to macrophages, raising the levels of microRNA-106b-5p in these cells. This increase triggers the PI3Kγ/AKT/mTOR signaling pathway by inhibiting programmed cell death 4 at the post-transcriptional stage. As a result, macrophages polarize into the M2 phenotype, which then enhances EMT-driven migration, invasion, and metastasis of CRC cells, establishing a positive feedback loop that accelerates cancer progression [84].

Quantitative proteomic analysis has identified upregulation of CD90 and EphA4 during the EMT process, which mediates physical interactions between cancer stem cells (CSCs) and TAMs by directly binding to their corresponding counter-receptors on these cells. Activation of EphA4 receptors on cancer cells further stimulates Src and NF-κB signaling pathways [85].

TAMs play a crucial role in facilitating the EMT process by secreting various cytokines and proteins. For instance, TAM-secreted IL-8 triggers the JAK2/STAT3/Snail pathway, inducing EMT in hepatocellular carcinoma cells [86, 87]. In oral squamous cell carcinoma, M1-like TAMs enhance EMT by increasing the secretion of IL-6, which also induces cancer stem-like transformations by upregulating MMP14 and MME expression in OSCC cells [88]. Similarly, TAMs promote migration and invasion of osteosarcoma cells and induce EMT via the STAT3 pathway by upregulating COX-2 and MMP9 [89]. M2 macrophages contribute to the EMT process by elevating transcript levels of EMT markers such as waveform protein and Snail, while downregulating E-calmodulin, critical for detaching cancer cells from surrounding tissues and enabling metastasis. Furthermore, M2 macrophages and CCL22 synergistically activate Snail in EMT through the SMAD signaling pathway, enhancing tumor invasion [90].

Additionally, co-culture with TAM led to EMT, elevated MMP-9 expression, and increased invasiveness in breast epithelial cells. Comparative proteomics analysis revealed that endoplasmic reticulum oxidoreductase (ERO1-α) was significantly elevated in breast epithelial cells co-cultured with TAM compared to those cultured alone. This increase was crucial for the TAM-induced invasive behavior and MMP-9 upregulation. Cytokine array analysis showed higher levels of interleukins and growth factors, indicating that CCL2 secreted by TAM promotes ERO1-α expression and enhances the invasiveness of breast epithelial cells [91].

Analysis of scRNA-seq data revealed higher proportions of immune cells, including macrophages and T cells, in mesenchymal-like (MES-like) tumor cells compared to neural-progenitor-like (NPC-like) tumors. A mouse glioblastoma transplantation tumor model and IHC assays showed a higher proportion of macrophages in MES-like tumor cells, confirmed by similar findings in human glioma samples. Treatment with chlorophosphate, which depletes macrophages, led to a significant reduction in MES-like glioma cells, illustrating a macrophage-induced transition to a mesenchymal-like state [92].

At metastatic sites, predominantly M2 subtype TAMs secrete IL-35, which promotes metastatic colonization by reversing EMT in cancer cells through JAK2-STAT6-GATA3 signaling. In primary tumors, EMT-induced expression of IL12R-β2, a subset of the IL-35 receptor, contributes to the response of cancer cells to IL-35 during metastasis [93]. Clinically, CD163 + TAMs infiltrating the tumor front are associated with EMT and poor prognosis in CRC patients. CRC-regulated macrophages regulate the EMT program and enhance migration and infiltration of CRC cells by secreting IL6, which activates the JAK2/STAT3 pathway and suppresses the tumor suppressor miR-506-3p in CRC cells. This miRNA is a crucial regulator of FoxQ1, which is down-regulated in CRC cells, leading to increased CCL2 production, promoting further macrophage recruitment [94].

In summary, TAMs and EMT often co-localize, creating a bidirectional relationship where EMT promotes macrophage recruitment and polarization, and M2 macrophages, in turn, enhance the EMT process, collectively driving tumor progression. This interaction highlights the complexity of the tumor microenvironment and highlights potential therapeutic goals. By disrupting the signaling pathways and interactions between TAMs and EMT cells, it may be possible to hinder tumor growth, reduce metastasis, and overcome resistance to current cancer treatments. Targeting these interactions offers promising avenues for developing more effective cancer therapies.

### ECM remodeling: The role of extracellular matrix alterations in modulating TAM function

CAFs are a crucial component of the TME, performing multiple functions such as regulation of ECM remodeling, metabolism, angiogenesis, and facilitating interactions with cancer cells and infiltrating immune cells through the secretion of growth factors, cytokines, and chemokines [95].

Many studies have noted the co-localization of CAFs and TAMs, which promotes ECM remodeling through intercellular interactions, resulting in an ECM that is significantly different from that of normal tissues—more rigid and fibrotic, creating an immunosuppressive environment. These alterations in the ECM facilitate tumor cell growth, survival, invasion, and can impede drug penetration, enhancing tumor metastasis [15, 96]. In breast cancer, CD163 + and CD206 + M2-like TAMs have been observed in very close proximity to αSMA + CAFs [97]. Similar observations of close spatial proximity between macrophages and CAFs have been made in the colon cancer TME, suggesting potential paracrine interactions that could influence cellular status [78].

TAMs not only co-localize with CAFs but also induce polarization of TAMs, contributing to ECM remodeling, tumor immunosuppression, and poorer prognoses. For instance, multi-omics techniques have identified a spatial ecological niche in hepatocellular carcinoma composed of SPP1 + macrophages and CAFs near the tumor border, which has been observed to stimulate ECM remodeling and promote the tumor immune barrier structure formation, thus limiting immune infiltration into the tumor core [98].

CAFs also facilitate the recruitment and differentiation of monocytes into M2-type macrophages by secreting monocyte chemotactic protein-1, SDF-1, and chitinase 3-like 1, which impairs effector T-cell responses and induces immunosuppression in the TME [15, 99]. They release CSF1 to enhance macrophage activities, and macrophages provide the ligand for PDGF receptor to support fibroblast survival [97].

In colorectal cancer, CAFs promote M2 macrophage polarization and recruitment by upregulating VCAM-1 expression in CRC cells and secreting chemokines IL-6 and IL-8/CXCR2 pathways, while also inhibiting NK cell function [100]. Newly identified extracellular matrix CAFs (eCAFs), located in the ECM, express osteopontin and have been shown experimentally to recruit M2 macrophages through enhanced periostin expression [101]. Additionally, Slit2 protein secreted by CAFs increases M1-type macrophage recruitment to tumors and enhances their capacity to phagocytose tumor cells both in vitro and in vivo. Slit2 also increases the expression of matrix metalloproteinases in M1-TAMs, which attenuates fibrosis in a mouse model of breast cancer.High expression of Slit2 correlates with improved survival and is negatively correlated with the density of CD163 + TAMs in patient samples [102].

Furthermore, studies have highlighted that ECM components, such as fibronectin, laminin-10, and multifunctional proteoglycans, modulate macrophage phenotypes, influencing their inflammatory responses [14, 103]. Decellularized ECM from human colorectal tumors has been shown to induce a relatively anti-inflammatory phenotype in macrophages, with increased expression of IL-10 and TGFβ and decreased expression of CCR7, TNFα, and IL-6 [104].

Stromal TAMs, characterized by higher production of CCL18, which is correlated with increased metastasis and poorer survival in breast cancer patients [105].TAMs also secrete a range of proteases that degrade the ECM, promote the infiltrative growth of tumor-associated blood vessels, and the mobilization of pro-angiogenic growth factors stored in the perivascular ECM. These macrophage-derived proteases include MMPs, such as MMP2, MMP9, and MMP12, and serine or cysteine proteases like histones and fibrinogen activators.

TAMs are abundantly present at the collagen-rich borders of tumors and play an important role in the deposition and organization of the collagen ECM. TAMs express the mRNAs and proteins of P4HA1, PLOD1, and PLOD3, essential for collagen maturation. In Ccr2-/- collagen I tumors, P4HA1 and PLOD3 protein expression is notably reduced, underscoring the key role of TAMs in ECM dynamics and tumor progression [106].

In conclusion, TAMs and CAFs interact to promote ECM remodeling and tumor invasion. As both CAFs and TAMs are abundant in the TME, targeting their interactions might enhance antitumor therapeutic efficacy, rather than targeting either cell type alone. This combined approach could improve outcomes by disrupting the tumor-supportive microenvironment and mitigating drug resistance.

### Intravasation: differences observed between TAMs near blood vessels and those distant from vessels

Cancer metastasis is a multifaceted and dynamic process, where tumors, having established a TME conducive to growth through angiogenesis, inflammation, EMT, and ECM remodeling, can invade the bloodstream or lymphatic system to achieve widespread metastasis [66]. The entry of tumor cells into the bloodstream is intricate, with TAMs playing a pivotal role. As previously discussed, during tumor angiogenesis, recruited TIE2 + TAMs disrupt the integrity of tumor vasculature, increase vascular leakage, and facilitate cancer cell endocytosis [37].

During intravascular infiltration, macrophages localize to the perivascular microenvironment, assisting cancer cells in traversing the vascular barrier and navigating through the metastatic TME portal. CCR2 signaling orchestrates the recruitment of motile TAMs, which then differentiate into sessile perivascular macrophages. CXCL12, released by perivascular fibroblasts, lures motile TAMs and cancer cells toward the vasculature. Once they reach the vasculature, these TAMs differentiate into perivascular macrophages, which enhances vascular leakage and supports endocytosis [107].

Moreover, macrophages are directly involved in the endocytosis of tumor cells. They activate RhoA signaling in cancer cells, inducing the formation of invasive protrusions and subsequent endocytosis in vitro [108]. Additionally, TAMs can transform tumor cells into a stem cell-like state, enhancing their invasiveness and endocytosis capabilities through Notch-Jagged signaling [1].

### Extracellular vesicles: the involvement of communication via extracellular vesicles in shaping TAM characteristics

Exosomes are the tiniest extracellular vesicles (EVs), 30 to 150 nm in diameter, that play a key role in cell-to-cell communication. These vesicles contain mainly proteins and RNA, but may also contain sugars, lipids, and DNA. exosome proteins include intact membrane proteins, peripheral surface proteins, endosomal proteins, and enzymes [109]. Exosomes significantly influence TAM and tumor cell interactions and are instrumental in TAM recruitment during tumor progression. They are secreted into the TME to modulate the functions of nearby cells, thereby fostering an environment favorable for tumor cell growth.

Specific proteins within exosomes, such as CSF1, CCL2, and EMAP2, are known to mediate macrophage recruitment and M2 polarization [110]. Various types of RNA within exosomes secreted by different cells can be absorbed by TAMs through multiple pathways, altering the status and function of TAMs. For example, in HCC, the exosome Sal-like protein 4 bound to the promoter region of miR-146a-5p and up-regulated its expression in HCC-derived exosomes, thereby promoting M2 polarization and suppressing T cell function [111, 112]. Liver tumor-derived long non-coding RNAs (lncRNAs) like TUC339, circular RNAs (circRNAs) like hsa_circ_0074854, and microRNAs (miRNAs) like miR150 are considered key signaling mediators orchestrating macrophage M1/M2 polarization [111].

In ovarian cancer, exosome-derived miR222-3p from cancer cells acts as a potent regulator of M2 polarization, enhancing cancer progression [113]. In HCC, exosome miR-23a-3p decreases PTEN expression, leading to increased levels of phosphorylated AKT and PD-L1 in macrophages. This suggests a regulatory mechanism driven by HCC cell-derived exosomes via the miR-23a/PTEN/AKT pathway [114]. In pancreatic cancer, exosome miR-301a-3p, associated with hypoxia, induces M2 macrophage polarization by suppressing PTEN and activating the PI3Kγ signaling pathway [115]. Hypoxic conditions in lung cancer drive the release of EVs that increase M2 phenotype polarization through the transfer of miR-103a. This miRNA leads to reduced PTEN levels, thereby increasing AKT and STAT3 activation along with the expression of various immunosuppressive and pro-angiogenic factors. Conversely, inhibiting miR-103a reduces M2-type polarization and boosts cytokine production in tumor-infiltrating macrophages, demonstrating the feedback loop that enhances cancer progression and tumor angiogenesis [116].

In colorectal cancer, miR-195-5p is dramatically under-regulated in tissues and is associated with poor prognosis. Alterations in miR-195-5p in colon cancer cells lead to notable changes in migration and EMT. Mechanistically, miR-195-5p regulates NOTCH2 expression post-transcriptionally by binding directly to the 3’-UTR of Notch2 mRNA, followed by miR-195-5p/NOTCH2 inhibition of GATA3-mediated IL-4 secretion, ultimately influencing M2-like TAM polarization in CRC cells [117]. Several miRNAs, such as miR-25-3p, miR-130b-3p, and miR-425-5p, can be transferred from CRC cells to macrophages via exosomes. These exosomal miRNAs regulate PTEN by activating the PI3K/Akt signaling pathway, leading to M2 macrophage polarization and promoting CRC liver metastasis at the invasive front [118].

Tumor-derived exosomes, a subclass of EVs, are taken up by macrophages and induce macrophages to express PD-L1, enhancing their immunosuppressive capacity [119]. The lncRNA BCRT1 can bind competitively to miR-1303, preventing the degradation of PTBP3, a gene that promotes tumor growth in breast cancer. Overexpression of lncRNA BCRT1 promotes exosome-mediated macrophage M2 polarization, thereby accelerating breast cancer progression. Additionally, lncRNA BCRT1 is upregulated in hypoxic regions due to the binding of HIF-1α to the HRE in the promoter of lncRNA BCRT1 [120].

The oncogene SERPINE2-derived circRNA, named cSERPINE2, functionally shuttles to TAMs via tumor exosomes and enhances the secretion of IL-6, leading to increased proliferation and invasion of breast cancer cells. IL-6, in turn, in a positive feedback mechanism, boosts intra-tumoral EIF4A3 and CCL2 levels, further enhancing tumor cSERPINE2 biosynthesis and promoting TAM recruitment [121].

EVs are crucial in cancer progression, as demonstrated by the osteosarcoma Rab22a-NeoF1 fusion protein and its chaperone PYK2. HSP90, via its KFERQ-like motif (RVLFLN142), sorts these components into exosomes. Within exosomes, the Rab22a-NeoF1 fusion protein helps form pre-metastatic lung niches by attracting bone marrow-derived macrophages. Furthermore, exosomal PYK2 activates RhoA in receptor-negative osteosarcoma cells and triggers STAT3 in receptor-positive macrophages, promoting the M2 macrophage phenotype [122].

Studies with homozygous mouse models and cancer patient samples have revealed that TME cytokines attach to tumor-derived exosomes via the glycosaminoglycan (GAG) side chains of proteoglycans. These cytokine-bound exosomes are preferentially absorbed by cytokine receptor-positive cells, altering the immune landscape of these tissues and increasing the metastatic spread of cancer [123].

TAMs enhance aerobic glycolysis and resistance to apoptosis in breast cancer cells through the delivery of the bone marrow-specific lncRNA, HIF-1α stable long non-coding RNA (HISLA), via EVs. HISLA inhibits the interaction between PHD2 and HIF-1α, preventing HIF-1α degradation. This interaction is enhanced by lactate released from glycolytic tumor cells, creating a feed-forward loop that promotes cancer progression [124]. Additionally, M2 macrophage-derived exosomes highly express miR-21-5p and miR-155-5p, which facilitate migration and invasion of colorectal cancer cells by binding to the BRG1 coding sequence and downregulating its expression [125].

Furthermore, the circular RNA circ_0020256 in TAM-secreted exosomes promotes proliferation, migration, and invasion of cholangiocarcinoma cells. This promotional activity is mediated through the circ_0020256/miR-432-5p/E2F3 regulatory axis [126].

### Host cells: interactions between TAMs and other host cells within the tumor microenvironment

The recruitment and modulation of TAMs by tumor cells are pivotal for cancer progression. CD163 + and CD206 + TAMs are particularly enriched at the tumor invasion front, correlating with vascular density, whereas CD68 + cells show no regional differences. In metastatic melanoma, round melanoma cells near blood vessels and CD206 + TAMs with elevated CD206 mRNA levels are noted. Amoeboid melanoma cells induce M2 macrophage polarization via myosin II [127].

The multi-copy T-cell malignancy 1 stimulates IL-6 secretion, promoting M2-like macrophage polarization and enhancing Triple-negative breast cancer cell invasiveness. MCT-1 also increases soluble IL-6 receptor levels, with IL-6R antibody antagonizing these effects [128].

Tumor cell-derived spondin 2, an ECM glycoprotein, plays a complex role in macrophage and neutrophil recruitment during inflammation. It activates the integrin-PYK2 pathway in monocytes/macrophages, enhancing their transendothelial migration and infiltration into colorectal cancer. However, in hepatocellular carcinoma, SPON2 promotes the infiltration of M1-like macrophages while inhibiting tumor cell migration and metastasis, highlighting differential impacts based on cancer type and TME [129].

Epithelial membrane protein 3 is linked with immunosuppression in GBM. Elevated EMP3 levels in GBM are associated with high PD-L1 expression and extensive M2 TAM recruitment, leading to suppressed T-cell infiltration and effective responses to anti-PD1 therapy [130].

The CCL2-CCR2 axis activation within the TME is known to promote tumor angiogenesis and recruit TAMs and myeloid-derived suppressor cells (MDSCs) in various cancers, including sarcoma and breast cancer. The deletion of RB enhances fatty acid oxidation through AMP-activated protein kinase (AMPK) activation, leading to increased mitochondrial superoxide production and JNK activation, which boosts CCL2 expression [131].

A subpopulation of TAMs expressing folate receptor β (FRβ) shows M2-like characteristics of immunosuppression. In a homozygous tumor mouse model, chimeric antigen receptor (CAR) T cell-mediated specific clearance of FRβ + TAMs enriches pro-inflammatory monocytes, increases the influx of endogenous tumor-specific CD8 + T cells, and delays tumorigenesis and prolongs survival [132].

Intraductal papillary neoplasms (IPN), precancerous lesions of cholangiocarcinoma, show a significant reduction in CD8 + T and CD20 + B lymphocyte counts compared to biliary epithelial neoplasms (BilIN). As biliary dysplasia progresses to an invasive state, CD68 + and CD163 + macrophage infiltration increases in the tumor mesenchyme rather than the epithelium, indicating temporal heterogeneity in immune cell distribution within the TMEs from different origins [133].

CSCs are crucial for monocyte recruitment across tumor types; supernatants from cholangiocarcinoma, hepatocellular carcinoma, or glioblastoma cells under CSC-enriched conditions show elevated levels of tumorigenic macrophage factors like CCL2, CCL5, CSF1, GDF15, IL-13, TGFβ, periostin, and WISP1. These factors, produced exclusively by CSCs, particularly periostin, are secreted preferentially by CSCs in glioblastomas and cholangiocarcinomas to enhance TAM recruitment [134, 135]. TAMs promote CSC self-renewal and tumor progression via various inflammatory factors and EMT. For instance, in breast and colon cancers, mesenchymal stem cells (MSCs) foster CSC ecological niche formation by secreting prostaglandin E2 (PGE2), IL-6, IL-8, and CXCL1 [136].

### Other immune cells: the cross-talk between TAMs and other immune cell types influencing TAM heterogeneity

#### TAM and T cell dynamics in cancer progression

Studies highlight significant co-localization of TAMs and T cells within the TME. Specifically, FOLR2 + macrophages, located near blood vessels within the tumor stroma, have been observed aggregating with CD8 + T cell clusters. This interaction is linked to favorable clinical prognostic outcomes, underscoring the anti-tumor potential of this macrophage subpopulation and its beneficial implications for cancer therapy [137]. Further analysis of the tumor beds and stromal regions of patients shows that PD-L1 + macrophages are prevalent, particularly in T cell-rich areas, indicating a symbiotic relationship between these macrophages and the immune cells within the TME [138]. In metastatic clear cell renal cell carcinoma, in situ observations reveal co-localization between M2 macrophages and depleted CD8 + T cells, supporting the interaction between these two immune groups in the later stages of the disease [35].

Differential gene analysis of monocyte subpopulations in gallbladder cancer with ErbB mutations shows elevated expression of STAT1, CXCL9, and CXCL10 in M2 macrophages. This expression profile facilitates stronger interactions between M2 macrophages and CD4 + T cells, including Tregs, suggesting that M2 macrophages play a role in recruiting or activating CD4 + T cells and Tregs, thereby aiding tumor cells in evading the immune response. Notably, the MDK-LRP1 pathway has been identified as a critical factor in promoting the directed differentiation of macrophages into the M2 phenotype. Experimental evidence confirms that MDK-induced M2 macrophages can recruit Tregs, contributing to immune escape mechanisms. IHC validation shows that MDK is significantly overexpressed in GBCs with ErbB pathway mutations, correlating with poorer patient survival [139]. These findings highlight the complex interplay between macrophages and T cells within the TME, emphasizing the critical roles these interactions play in cancer progression and potential therapeutic strategies.

#### TAM and T cell interactions in promoting M2 polarization

Various T cells, including CD8 + T cells, Tregs, and follicular helper T (TFH) cells, play crucial roles in recruiting and influencing the polarization of TAMs through distinct pathways.

**CD8 + T Cell** The depletion of CD8 + T cells, a hallmark of tumor immunosuppression, is influenced by exhausted T cells(Tex)-expressing factors which recruit monocytes to the TME and cause them to differentiate into macrophages. These macrophages develop unique long-term interactions and synapse formations with CD8 + T cells, further promoting immunosuppression [140]. Interactions between depleted CD8 + T cells and M2-like TAMs involve cytokine and chemokine signaling and cell adhesion, focusing on T-cell suppression and M2-like polarization of macrophages. M2-like TAMs express various ligands for T-cell immune checkpoints, such as PD-L1 (binding to PD-1), CD80 and CD86 (binding to CTLA4), NECTIN2/CD112 and PVR/CD155 (binding to TIGIT), LGALS9/Galectin-9 (binding to TIM-3), TNFRSF14/HVEM (binding to BTLA), and SPP1 (binding to CD44), thereby enhancing the immunosuppressive tumor microenvironment [35].

**Tregs** TAMs in HCC are known to attract Tregs, significantly impacting the TME. The presence of FoxP3 + Tregs correlates with a high density of TAMs. The application of anti-IL 10 antibodies has been shown to partially inhibit this correlation, suggesting TAM-driven Treg expansion contributes to HCC progression [141, 142]. In addition, TREM-1 + TAMs also respond to hypoxic conditions and tumor metabolites through the ERK/NF-κβ pathway, resulting in an increase in CCR6 + Foxp3 + Tregs, which promotes tumor immunosuppression and creates resistance to PD-L1-targeted therapies [142, 143].

In experimental settings, Tregs have been observed to enhance macrophage expression of M2 markers such as CD163 and PDL1 while reducing TNFα expression, indicating that Tregs may regulate macrophage function through the interaction of HLA and LILRB1. Blocking this interaction could potentially amplify anti-tumor immunity, especially in esophageal squamous cell carcinoma [144]. Treg cells also inhibit IFN-γ secretion by CD8 + T cells, thereby preventing the activation of fatty acid synthesis in immunosuppressive M2-like TAMs and promoting their survival and metabolic adaptation [145].

CCL22 levels are notably higher in malignant pleural effusions (MPE) than in non-malignant ones, strongly correlating with poor survival in lung cancer patients. Produced primarily by TAMs, CCL22, mediated by TGF-β through c-Fos, plays a crucial role in recruiting Tregs in MPE. Subsequently, the high level of IL-8 secreted by Treg further induces TGF-β production by TAMs and promotes immunosuppressive TME in MPE [146].

**Others** In HCC, newly identified T follicular helper (T_FH_) cells exhibiting a CXCR5-PD-1-BTLA-CD69high phenotype promote the conditions for M2b macrophage polarization, enhancing the tumor’s capacity to evade immune responses [147]. IL-13 derived from malignant epithelial cells or TH2-polarized CD4 + T cells and CD1d-restricted natural killer T (NKT) cells may promote the pro-tumorigenic effects of TAMs through activation of the STAT6 signaling pathway, but this has not been demonstrated in vivo. [148].

#### Multifaceted roles of TAMs in the tumor immune microenvironment (TIME)

Early TAMs co-express a variety of T cell co-inhibitory and co-stimulatory receptors. CD14 + cells within tumors and distant lung tissues significantly elevate the expression of these molecules compared to peripheral blood CD14 + monocytes, indicating a high degree of heterogeneity but consistent upregulation in the tumor context [149]. Among macrophages, M2 TAMs as well as TAMs expressing AREG and CXCL10 are the main cells that interact with T cells. In contrast, macrophage-CXCL10 and macrophage-C1QC-PLTP may send richer signals to T cells and NK cells, while CTLA4 + Treg is predicted to receive more signals from macrophages [150]. In TME, M2-polarized macrophages facilitate the transformation of CD4 + CD25- T cells into adaptive Tregs (aTregs). In turn, these resulting aTreg cells induce monocytes to differentiate into TAMs. This M2 macrophage-aTreg cell positive feedback cycle contributes to immunosuppression and has been associated with advanced clinical stage and poorer survival in patients [151].

TAMs can suppress effective adaptive immunity through various mechanisms. They enhance the function of Tregs, induce T cell starvation via metabolic pathways, and trigger the expression of suppressive immune checkpoints [150]. A specific TAM subpopulation identified as PLTP + C1QC + TAM regulates the number of dysfunctional T cells through cytokine and chemokine signaling [150]. Additionally, TAMs directly inhibit CD8 + T cell proliferation by metabolizing L-arginine via arginase 1, ,oxygen radicals, iNOS or nitrogen species [18].

Co-culture experiments using the Transwell system demonstrated that hepatic CD163 + macrophages inhibit mucosa-associated invariant T cell (MAIT) function through cell contact and PD-L1-dependent mechanisms. Flow cytometry revealed specific depletion of the hepatic F4/80 high CD11bint macrophage population, and its exhaustion led to a remarkable increase in tumor-infiltrating MAIT cells and increased expression of IFN-γ, TNF-α, and granzyme B [152].

In the context of melanoma, MDMs trigger glucocorticoid signaling that activates an array of checkpoint receptors including PD-1, Tim-3, LAG-3, and IL-10, and reduces levels of pro-inflammatory cytokines such as TNF-α, IL-2, and IFN-γ. This modulation maintains the dysfunctional state of CD8 + T cells, thereby diminishing the effectiveness of immunotherapy [153].

#### The roles of B cells in TAM dynamics

B cells play a critical role in influencing the pro-carcinogenic functions of TAMs. They promote the polarization of M2b macrophages in HCC and suppress other immune cells in the TME, such as CD8 + T cells and M1 macrophages, thereby facilitating cancer cell proliferation. Depleting B cells can prevent M2b production, enhance anti-tumor T cell responses, and reduce tumor growth [18].

**Regulatory B cells (Bregs)** In metastatic breast cancer mouse models, Bregs producing TGFβ have been observed to bias macrophages toward an immunosuppressive M2 phenotype. In addition, transfer of B cells or serum from tumor-immunoreactive mice to Rag1-/- mice increases the infiltration of tumors by both innate and pro-inflammatory immune cells. These B cells are transformed into antibody-producing cells that generate IgG, which activates macrophages with tumor-promoting inflammatory effects that express the Fcγ receptor (FcγR), leading to chronic inflammation in precancerous and malignant lesions [154].

**Plasma cells** B cells also critically regulate the microenvironment of HCC and colorectal cancer liver metastases (CRLM). Single-cell analysis showed that IgG + plasma cells were recruited by TAMs through the CXCR3-CXCL10 axis and were enriched in hepatocellular carcinomas. In contrast, IgA + plasma cells were recruited by metastatic tumor cells mainly in CRLM through CCR10-CCL28 signaling, and were mainly present in CRLM. Functionally, IgG + plasma cells promote pro-tumorigenic macrophage production, whereas IgA + plasma cells in CRLM induce MDSC activation [155].

**Tumor-infiltrating B-lymphocytes (TIL-Bs)** In metastatic ovarian tumors, TIL-Bs secrete GM-CSF, IFNγ, IL-12p40, CXCL10, and IL-7, which stimulate macrophages, T cells, and dendritic cells (DCs) [156, 157]. Plasmablast-like TIL-Bs from patients with ovarian cancer and melanoma show elevated levels of transcripts for IFNγ and chemokines like CCL3, CCL4, and CCL5, which attract T cells, macrophages, and NK cells, leading to increased T cell infiltration [157]. Conversely, B-cell-derived GABA promotes the differentiation of monocytes into anti-inflammatory macrophages that secrete IL-10 and inhibit CD8 + T cell activity [158].

**Tertiary lymphoid structures (TLS)** In colorectal cancer (CRC), tumors lacking CXCL13 have fewer intratumoral B cells and a worse prognosis than those expressing CXCL13. The interaction between depleted or dysfunctional CD8 + and CD4 + tumor-infiltrating lymphocytes (TILs) and the B-cell recruiting ligand CXCL13 leads to the formation of tertiary lymphoid structures (TLS). Analysis in CRC also shows a significantly higher frequency of CD4 + Foxp3 + Treg cells and CD68 + CD163 + M2 macrophages in intratumoral TLS compared to peritumoral TLS, suggesting a potential correlation between peri-tumor TLS and the immunosuppressive environment within the tumor [159].

#### The roles of innate immune cells and MDSCs in TAM dynamics

In melanoma, MARCO-expressing macrophages undergo major changes in metabolism while activating NK cell killing of tumor cells via TNF-related apoptosis-inducing ligands. This effect differs from previous studies because macrophages traditionally inhibit NK cell activation. When it is combined with T cell-targeted immunotherapies such as PD-1 or PD-L1 antibodies, this synergistic effect greatly enhances tumor cell eradication [160].

In prostate cancer (PCa), CD1d-restricted invariant natural killer T (iNKT) cells play a crucial role in modulating the TME. In a mouse prostate cancer model, iNKT cells inhibit the growth and immunosuppressive effects of tumor-infiltrating immune cells. They did this by reducing the presence of TIE2 + M2-like macrophages, which induce tumor angiogenesis, and pro-inflammatory M1-like macrophages. iNKT cells slowed tumor progression by making direct contact with macrophages within the PCa base and by strategically transferring iNKT cells into tumor-bearing mice. Through interactions involving CD1d, Fas, and CD40, iNKT cells promote selective clearance of M2-like macrophages while retaining M1-like macrophages, which has been associated with reduced invasiveness and decreased expression of pro-angiogenic genes in human PCa [161].

MDSCs represent a heterogeneous population originating from bone marrow, acting as precursors to DCs, macrophages, and granulocytes. MDSCs significantly suppress immune responses, and their recruitment or induction of immune-productive cell death leads to an increase in inflammatory TAMs. Conversely, inhibition of glutamine metabolism within MDSCs triggers activation-induced cell death and converts them into inflammatory macrophages, suggesting a complex but targeted interaction within the immune system [162].

Additionally, TAMs that express CD39 and CD73 enhance the infiltration of both MDSCs and TAMs, creating a self-amplifying loop that facilitates local immune escape, underscoring the interconnected and dynamic nature of immune responses within tumors [163].

#### Not all macrophages are enemies

The CXCR3 receptor, expressed by CD8 + and CD4 + T cells, and its ligands CXCL9, CXCL10, and CXCL11, predominantly produced by DCs and CD169 macrophages, are involved in mechanisms that promote immune cell infiltration into tumors, particularly those that are immune-excluded or at the immune margin [164].

TIM4 is abundantly expressed on macrophages in the T-cell regions of cancer-associated TLSs. This expression is positively correlated with markers for B cells, effector CD8 + T cells, and a chemokine signature characteristic of TLS, suggesting a role in protective immunity [165].

CD169 + macrophages, predominant in tumor-draining lymph nodes, proliferate and expand in response to tumor stimuli. Depleting these macrophages increases lung metastasis. The expansion of CD169 + macrophages correlates with B-cell expansion in these lymph nodes, and B-cell depletion negates the anti-metastatic effects of CD169 + macrophage deficiency, highlighting a protective role for CD169 + lymph node macrophages in breast cancer metastasis [166].

## Accurate detection strategy for spatiotemporal heterogeneity

The development of advanced genomic technologies has significantly enhanced our understanding of the heterogeneity within tumors and macrophage populations. By employing mIHC and spatial transcriptomics, researchers can now analyze cellular variations across different regions of tumors more effectively. This discussion highlights several contemporary methods and their associated challenges.

Single-cell RNA sequencing (scRNA-seq) is pivotal for analyzing the transcriptome of cells within the TIME. These techniques provide high-resolution, unbiased analyses of various cell types, including tumor cells, myeloid cells, T cells, and mesenchymal cells, illuminating the immune heterogeneity across tumor types [6]. Single-cell technologies can be divided into two main groups: “targeted” technologies (FACS, CyTOF, qPCR) and unbiased technologies (plate-based protocol, Fludigm C1, pooled approaches, and massively parallel approaches) [6]. Single-cell technologies vary widely in their scope—some offer broad coverage of cells but limited depth of gene expression per cell, while others, like plate-based protocols or massively parallel approaches, provide comprehensive gene expression insights [6].

Single-cell sequencing is particularly useful for understanding the spatiotemporal heterogeneity of TAMs. By analyzing data from different time points and tumor regions, researchers can track macrophage dynamics throughout tumor progression and their distribution within the TME. This method reveals the diverse nature of macrophages, including their functional states (e.g., M1 vs. M2), activity levels, proliferative capacities, and interactions with tumor cells, immune cells, and mesenchymal cells, among other aspects. For instance, scRNA-seq data from TAMs in MMTV-PyMT tumors were analyzed using QIAGEN Ingenuity Pathway Analysis software, which helped predict polarization signals specific to the LYVE-1 + TAM subpopulation [50]. However, scRNA-seq necessitates the dissociation of tissues into single cells, which eliminates spatial context. To compensate, spatial patterns of gene expression must be reconstructed from the original tissue coordinates of the sequenced cells [167]. This process is extremely complicated. In contrast, Spatial Transcriptomics sequencing is a technology that allows us to preserve the spatial location of the sample while using sequencing to obtain gene expression data for a more comprehensive and intuitive analysis of the organisation [31].

Spatial Transcriptomics is revolutionizing cancer biology by enabling the visualization and tracking of cancer subclones, detection of specific biomarkers, and examination of cellular interactions within tumors. Tools like Squidpy, stLearn, Spatial Experiment, Giotto, Seurat, and STUtility facilitate the analysis of multimodal data, revealing cell types, states, and spatial distributions [168]. This technique is invaluable for cancer diagnostics and prognostics, combining spatial and histopathology data to predict cancer gene markers and classify cells at high resolution.

Spatial transcriptomics can be generally divided into microdissection-based spatial transcriptomic technologies (Laser capture microdissection (LCMQ), etc.), in situ hybridization-based spatial transcriptomic technologies (smFISH, etc.), in situ sequencing-based spatial transcriptome technologies (STARmap, etc.), spatial barcoding-based spatial transcriptome technologies (ST and visium technologies, etc.), and other aspects [169].

Spatial multi-omics technologies have shown increasing promise in revealing the spatial distribution of TAMs. A study using spatial proteomic profiles of healthy and fat human and mouse livers, combined with single-nucleus sequencing, spatial transcriptomics, single-cell CITE-seq, and spatial proteomics, has analyzed the heterogeneity of lipid-associated macrophages (LAMs) and revealed their different spatially-resolved cellular ecological niches and associated influencing factors [170].

Moreover, technologies like spatially resolved transcript amplicon readout mapping detect multiple transcripts in tissue sections, offering high spatial resolution and the capability to produce 3D transcriptome images [168]. Integrating spatial and single-cell RNA sequencing data can map different cell types within tissues, infer cell compositions, and enhance the accuracy of spatial data through interpolation analysis. Spatial trajectory analysis can reconstruct patterns of cancer transformation and metastasis, combining spatial histology with histopathology images for validation and deeper insights into cell types and processes. Despite the important role of Spatial Transcriptomics in spatial location resolution, there are still problems such as relatively cumbersome process and low detection efficiency [169].

mIHC has made technical innovations in multilabel staining, spectral imaging and intelligent analysis, overcoming the technical deficiencies of gene expression profiling and flow cytometry, which are unable to obtain in-situ spatial information of proteins and cells. mIHC technology has its unsubstitutable and obvious advantages in analysing the TME. For example, the heterogeneity and distribution of TAMs in breast and gastric cancers were explored through fluorescence microimaging and a multiplex panel including markers like CD68, CD163, CD206, IRF8, and PDL1, identifying distinct TAM populations within tumor and adjacent tissues [22, 24]. This method provides a powerful approach for detailed cellular and molecular characterization in cancer research.

In conclusion, a variety of techniques can now be used to resolve spatial variations in TAMs. Spatial transcriptomics has become a widely used and promoted tool for establishing spatial maps, elucidating tissue structure, and conducting disease research. Its potential to reveal inter-cellular interactions at the spatial level is particularly promising [169, 171]. Additionally, single-cell sequencing technology has significantly advanced spatial transcriptomics by providing marker genes for cell typing, which in turn helps distinguish subpopulations using spatial location information [172]. Each technology has its own application areas and limitations, so we must effectively combine multiple methods to achieve our research objectives. Moving forward, leveraging the features of multiple technologies in combination can enhance our understanding of the spatio-temporal distribution and spatial mapping of TAMs and other cells. We anticipate that spatial multi-omics technologies will lead to more breakthroughs in research and provide valuable insights for deciphering dynamics of TAMs in cancer progression and developing therapeutic approaches (Table 1).

**Table 1:** The method of the spatiotemporal heterogeneity of tumor-associated macrophages (TAMs)

Method	Research filed	Cancer	Finding	References
scRNA-seq	Unravel macrophage functions in inflammatory TME	pancreatic cancer	An inflammatory cycle exists between tumor cells and IL-1β-expressing TAMs, which are caused by local synergy between PGE2 and TNF-expressing macrophage subpopulations in inflammatory TME.	[34]
scRNA-seq	Analyzing the dynamic communication between CAFs subsets and TAMs	Gastric cancer	CAFs interact with M2 macrophages by expressing periostin in regions enriched with ECM.	[101]
scRNA-seq and mIHC	Exploring spatiotemporal patterns of TAMs corresponding to vascular changes during glioblastoma progression	Glioblastoma	The hypoxic niche was found to attract and polarize TAMs, turning them into an immunosuppressed state.	[29]
scRNA-seq	Dissecting spatial cellular interaction networks within MAIT cell neighborhoods	Hepatocellular carcinoma	In hepatocellular carcinoma, the interaction between CSF1R(+)PD-L1(+) TAMs and MAIT cells promotes tumor progression in the tumor invasive margin.	[152]
spatial transcriptomics and mIHC	Analyzing the spatial distribution and clustering of macrophages	Lungs adenocarcinoma	The subtypes and distribution of TAMs are different in different lung adenocarcinoma subtypes.	[31]
ZipSeq(a spatial transcriptomics method)	Investigating the relationship between TAMs and T cell exhaustion in TME	Mouse models of melanoma (B78ChOVA, B16ChOVA) and spontaneous breast cancer	Exhausted T cells express factors that actively recruit monocytes to the TME and differentiate them into TAMs. TAMs and CD8(+) T cells spatially interact with exhausted T cells via synaptic interactions.	[140]
mIHC	Analyze the characterization of TAMs and their interaction with CAFs	Triple-negative breast cancer	It identifies a monocyte-derived STAB1 + TREM2 high-lipid-associated macrophage (LAM) subpopulation that is recruited to tumor tissue through the CAFs-CXCL12-CXCR4 axis and forms an immunosuppressive microenvironment.	[71]
mIHC	Investigate the heterogeneous relationship between tumor environment and TAMs	Gastric cancer	The study used a m-IHC panel including markers such as CD68, CD163, CD206, IRF8, and PDL1 to describe seven major TAM populations distributed between tumor and non-tumor tissues	[22]
scRNA-seq and mIHC	Exploring spatial heterogeneity of TAMs in glioblastoma	Glioblastoma	Specific markers such as IBA1, TMEM119, CXCL3 and TREM2 were used to distinguish between monocyte-derived TAM (Mo-TAM) and microglia-derived TAM (Mg-TAM). This approach identifies the spatial distribution of TAM subsets, particularly their enrichment in perivascular/necrotic regions or at the tumor-brain interface.	[33]
Zman-seq	Investigating Immune Cell State Shifts in the TME	Homozygous in situ mouse model of glioblastoma	Reveals progression of homing cytotoxic natural killer (NK) cells to state with low cytotoxic antitumor activity within 24 h and transformation of monocytes into immunosuppressive tumor-associated macrophages (TAMs)	[175]
scRNA-seq and ST analysis	Investigating the role of macrophages in suppressing the immunotherapeutic effects of liver metastases	Mouse model of liver cancer	Through analysis, this experiment presents a single-cell and spatial map of colorectal liver metastases and identifies specific subtypes of MRC1 (+) CCL18 (+) M2-like macrophages at the site of metastasis	[36]
Stereo-seq	Analyze the cellular distribution of invasive margins in patients with hepatocellular carcinoma	liver cancer	This experiment identified a subpopulation of damaged hepatocytes near the border next to the tumor with increased serum amyloid A1 and A2 (SAA) expression. Over-expression and secretion of SAA by hepatocytes in the invasion area may lead to the recruitment of macrophages and M2 polarization, further promoting local immune suppression and possibly leading to tumor progression.	[25]

CAFs, cancer-associated fibroblasts; TAMs, tumor-associated macrophages; ScRNA-seq, single-cell RNA sequencing; mIHC, multiplex immunohistochemistry; MAIT cells, mucosal-associated invariant T cells; TME, tumor microenvironment; IL-1β,interleukin-1β;PGE2, prostaglandin E(2) ;TNF, tumor necrosis factor

## Challenges for cancer therapy

With the in-depth study of the spatiotemporal heterogeneity of TAMs, new challenges and strategies for tumor therapy have emerged. The spatio-temporal heterogeneity of TAMs has significant implications for tumor therapy as TAMs exhibit different functions at different times and sites. For example, TAMs at the invasive front are more aggressive compared to TAMs in the tumor core, expressing factors like histones, and CD100, etc. that promote tumor invasion. This heterogeneity also impacts tumor responsiveness to drugs, where the role of TAM subpopulations in certain tumor regions limits the tumor’s response to treatment [14].

Research has demonstrated that the location and typing of TAMs contribute significantly to tumor drug resistance. TAMs enhance tumor proliferation and drug resistance through various mechanisms. For instance, IHC analysis of breast cancer biopsies has shown a high abundance of CCL18 + TAMs in patients resistant to chemotherapy, highlighting a positive correlation between the infiltration of CCL18 + TAMs and CD10 + GPR77 + CAF [97]. In addition, the spatial and temporal distribution and subtypes of TAMs are closely related to the prognosis of patients. For example, IL1B + TAMs found in the hypoxic zone of pancreatic cancer are associated with poor tumor prognosis [34].

We can assess the efficacy and prognosis of patients by monitoring changes in TAMs. Previously, treatments targeting TAMs inadvertently affected both M1 and M2 macrophages. Current strategies could benefit from targeting specific TAM subpopulations based on their distinct locations within tumors to achieve more precise treatment outcomes. Earlier sections have summarized the influence of multiple factors on the spatiotemporal distribution of TAMs and explored new strategies to leverage this heterogeneity in treatment approaches.

Current strategies for targeting TAMs mainly use TAMs-specific chemokine receptors, cytokines, etc., to block the recruitment of TAMs, reprogram TAMs, and change their function from pro-tumorigenic to tumor-suppressive [3]. Some of the very promising therapies include CSF1-CSF1R inhibitors, CCL2-CCR2 inhibitors, TREM inhibitors, and so on. CSF1R is a macrophage chemokine receptor that is present in all TAMs, whereas its ligand CSF1 can be produced by cancer cells and other components of the TME. By blocking this process, TAMs can be effectively prevented from entering the TME for therapeutic purposes [173]. The chemokine receptor CCR2 is mainly expressed by circulating monocytes, which can differentiate into TAMs after infiltrating into the tumor stroma. Studies have shown that blocking CCL2-CCR2 can effectively slow down tumor growth and invasion [20, 174]. TREM2 is predominantly found on certain specific macrophages, which can control the production of cytokines by macrophages, promote their migration and survival, and has been associated with a poorer prognosis of tumors [65, 175]. In a mouse model, targeting TREM2 effectively slowed tumor size and resulted in a better response to anti-PD-1 antibodies [176].

In addition to the therapeutic strategies described above, we have summarised targeting approaches for other factors. The hypoxic regions of tumors tend to accumulate TAMs alongside cytotoxic T cells, rendering them immunosuppressive. Therefore, targeting the tumor hypoxic microenvironment could enhance treatment efficacy. Studies have identified CCL8 and IL-1b as key factors in the recruitment and immunosuppression of TAMs in tumor hypoxia, suggesting that targeting these cytokines could improve treatment outcomes [29].

The interaction of TAMs with blood vessels significantly promotes tumor growth, proliferation, and migration. Targeting signaling molecules and pathways mediating these interactions could potentially improve tumor prognosis. For example, targeting the vascular endothelial RBPJ/CXCL2 axis may reduce the tumor-promoting effects of TAMs [9]. Strategies like co-targeting the EGFR and VEGFR signaling pathways and inhibiting the Notch signaling pathway to block oncogenic reprogramming may prove beneficial [177]. Furthermore, targeting specific TAM groups may inhibit tumor growth and enhance patient prognosis. SPP1 + TAMs and C1QC + TAMs, associated with tumor angiogenesis and phagocytosis respectively, highlight the potential of targeting distinct TAM subpopulations to influence immunotherapy effectiveness [62].

Inflammation is a critical driver of tumor growth, with the ability to recruit and polarize TAMs. Targeting inflammation-related factors or pathways that recruit TAMs may offer new therapeutic avenues. PGE2, a significant inflammatory mediator, has been shown to promote the accumulation of IL-1β + TAMs and monocytes, enhancing tumor growth. The use of NSAIDs, like aspirin and celecoxib, has been experimentally shown to reduce PGE2 levels and thus tumor progression [34, 75]. Ongoing clinical trials are assessing the effectiveness of dual EP2/EP4 antagonists (NCT04344795) and the potential of celecoxib to boost checkpoint inhibitor efficacy in CRC patients (NCT03026140 and NCT03926338). Additionally, targeting pro-inflammatory macrophages and blocking IL-1β signaling in tumor cells has been suggested to inhibit tumor growth [34] .

EMT is noted for increasing tumor drug resistance and reducing the efficacy of therapies. During EMT, tumor cells enhance TAM recruitment and polarization by secreting cytokines, microRNAs, and chemokines. Targeting specific molecules or TAMs involved in EMT could block this process and improve tumor prognosis. Current research suggests that macrophages may promote EMT through signaling pathways such as IL-35/JAK2-STAT6-GATA3, IL6/Jak/Stat3/THBS1, or STAT3/miR-506-3p/FoxQ1, which facilitate the invasion, and metastasis of tumor cells [88, 93, 94]. Targeting these pathways could yield new treatment strategies and facilitate the development of novel therapies.

It is also crucial to consider how interactions between CAFs and TAMs can promote tumor cell growth, survival, and invasion, as well as lead to ECM remodeling. The dense, remodeled ECM can hinder drug penetration, limit therapeutic efficacy, and contribute to tumor metastasis. Proposals to block interactions between SPP1 + macrophages and CAFs might enhance the effectiveness of immunotherapies. This is because SPP1 + macrophages and CAFs form an immunosuppressive spatial structure within the TME, which limits the infiltration of cytotoxic T cells [98]. Additionally, M2-type macrophages can activate the PI3K/Akt pathway through SPP1-CD44-mediated cell-to-cell interactions, promoting malignant cell proliferation and metastasis, thereby increasing recurrence and drug resistance in malignant gliomas [178]. This also provides new theoretical bases and potential targets for therapy.

During tumor progression, EVs play a significant role in TAMs and tumor cell interactions, as well as in TAM recruitment. Targeting related signaling pathways, including those mediated by exosomal ncRNAs from macrophages or tumor cells, is seen as a viable therapeutic strategy. Efforts to utilize M1 macrophage-derived exosomal ncRNAs as therapeutic targets are underway, with M1 macrophage-derived exosomes demonstrating high drug delivery efficiency [179]. Additionally, modifying how cytokines bind to tumor-derived exosomes could improve cytokine distribution within the TME [123].

Decreased CD8 + T cell infiltration in tumors is a negative prognostic marker. Macrophages and tumor-associated immunosuppression are closely linked. Interactions between macrophages and Tregs were found to contribute to the underlying immunosuppressive state. Therefore, treatments specifically targeting these pathways of immunosuppression may help to reactivate the anti-tumor immune response in esophageal squamous cell carcinoma [144]. Research involving CAR-T cells targeting folate receptor beta (FRβ) in tumor treatments has shown promising results, with potential improvements in tumor control and survival. Moreover, the combination of FRβ-specific CAR-T cells with conventional tumor-targeting CARs further enhanced the anti-tumor efficacy [132]. This opens new avenues for developing innovative therapies.

**Fig. 2:**
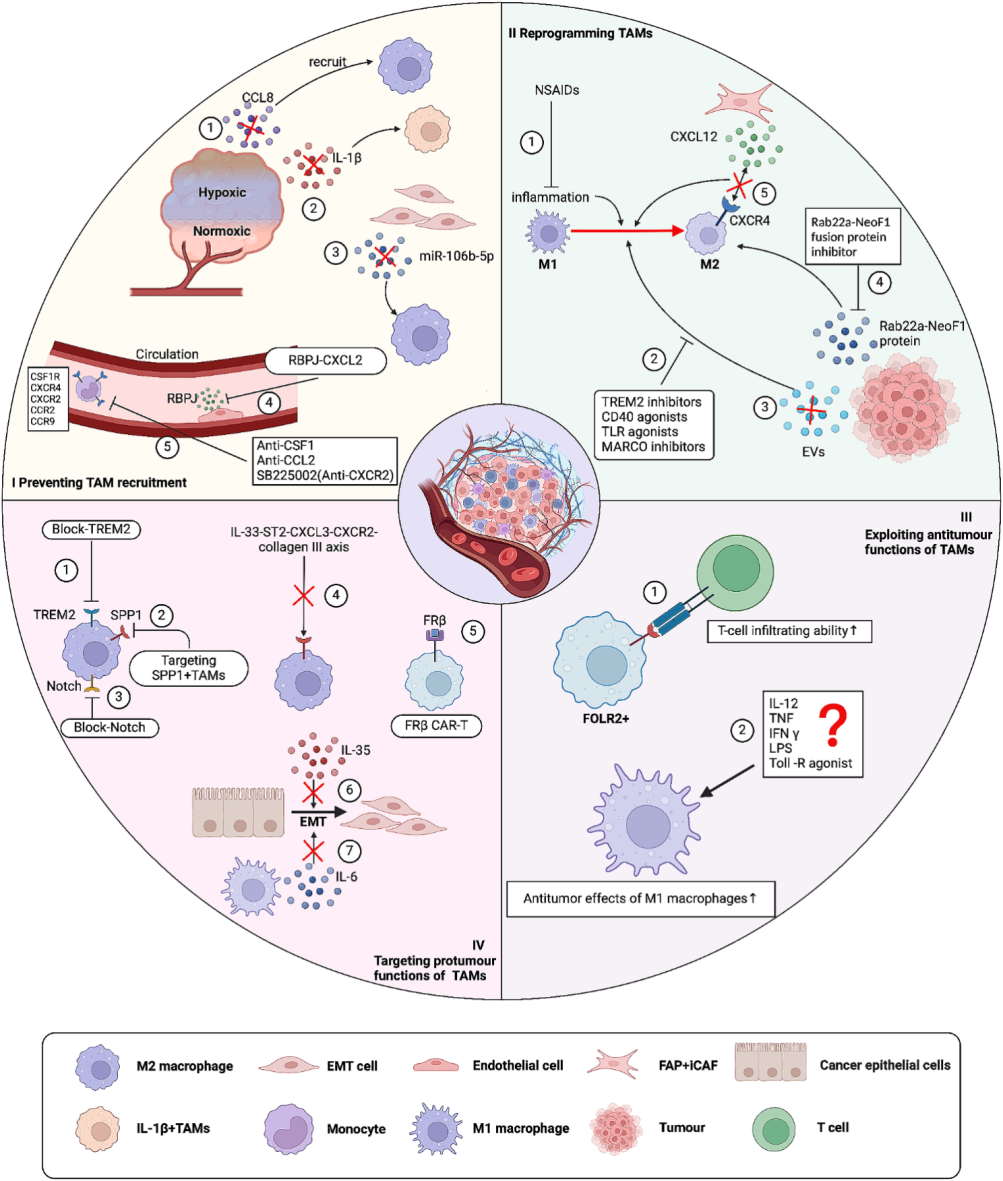
Therapies targeting tumor-associated macrophages. This figure illustrates the current strategies for targeting tumor-associated macrophages (TAMs) in cancer treatment. These strategies are categorized into four main approaches: (I) Blocking the recruitment of M2-type macrophages. This involves targeting the membrane receptors on macrophages and RBPj secreted by vascular endothelial cells to prevent TAMs to recruitment to tumor tissues. The hypoxic TME releases IL-1β, CCL8, while tumor cells undergoing EMT secrete miR-106b-5p, all of which can be targeted to hinder the recruitment of tumor-promoting macrophages and slow down tumor progression. (II) Reprogramming macrophages. Tumor inflammation triggers the release of CXCL12 by inflammatory cancer-associated fibroblasts (iCAFs) and the release of extracellular vesicles (EVs) and proteins by tumor cells, promoting the polarization of M1 macrophages into M2 macrophages. Targeting these molecules can prevent macrophage polarization and reprogram them. (III) Fulfill the function of tumor-suppressor macrophages. This strategy focuses on promoting the expression of specific molecules in tumor-suppressor macrophages to improve their anti-tumor functions. (IV) Targeting tumor-promoting macrophages. This includes blocking molecules on pro-tumorigenic macrophages and their associated secreted factors. It also involves receptor-specific chimeric antigen receptor (CAR)-T cell approaches to precisely kill or inhibit pro-tumorigenic macrophages

Despite the great promise, the use of TAMs to treat tumors still faces many challenges. As discussed previously, TAMs have different functions and phenotypes in different regions. This leads to difficulties in designing precise targeting of specific subsets of TAMs. In addition, the status of TAMs changes over time, which leads to the possibility that relevant therapeutic approaches may only be effective at a certain stage, thus reducing therapeutic efficacy [20]. Meanwhile, TAMs form complex interaction loops with multiple components of the TME, which may amplify or attenuate therapeutic effects. For example, M2-type macrophages promote malignant cell proliferation and metastasis with CAFs via SPP1-CD44-mediated cell-to-cell interactions, leading to increased tumor recurrence and drug resistance [178]. Trying to address these issues requires an in-depth understanding of the spatiotemporal heterogeneity of TAMs and their roles in TME in order to develop effective and precise tumor therapies. We believe that combining multiple analytical techniques can help us unravel the complex map of TAMs and bring breakthroughs in tumor therapy.(Table 2; Fig. 2).

**Table 2:** Potential therapeutic strategies targeting TAMs

Targeting	Drug name/potential targeting interventions	Cancer type	Clinical trial number	References
**hypoxia**	CCL8 and IL-1β and associated contexts in the hypoxic TME	Not specified		[29]
**Angiogenesis**	Vascular endothelial RBPJ/CXCL2 axis in TME;	CRC		[9]
	Inhibition of HMW-HA production by tumor cells;	CRC		[9]
	EGFR and VEGFR signaling pathways during tumor angiogenesis;	CRC		[9]
	Inhibition of Notch signaling pathway blocks onco-fetal reprogramming	HCC		[177]
	Anti-miR-155-5p and anti-miR-221-5p during tumor angiogenesis;	Pancreatic ductal adenocarcinoma		[61]
	Blocking TIE2 exosomal delivery or inhibiting the TIE2 pathway;	Cervical cancer		[54]
**Targeting specific TAMs**	Inhibition of VCAN+, INHBA+, and FN1 + TAMs subtypes	Not specified		[62]
**Inflammation**	Suppresses tumor inflammation and inhibits COX2 activity, including Aspirin, and Celecoxib;	Not specified		[11, 75]
	celecoxib (a cox-2 inhibitor);	CRC	NCT03026140, NCT03926338	
		Pancreatic		[34]
	TPST-1495(EP2 and EP4 antagonists);	CRC Stomach cancer	NCT04344795	
	ARY-007(EP4 antagonist);	Microsatellite stable CRC	NCT03658772	
	CXCL12-CXCR4 Axis in tumor cells	Breast Cancer		[71]
	Blocking IL-1β signaling in tumor cells	Pancreatic		[34]
	SB225002(a CXCR2 inhibitor)	Pancreatic		[72]
	Blocking the IL-33-ST2-CXCL3-CXCR2-collagen III axis	Pancreatic		[72]
	Liposomal co-delivery of aPKCι-siRNA and GEM (guicasibine)	Cholangiocarcinoma		[13]
**EMT cell**	Exosome miR-106b produced by tumor cells	CRC		[84]
**Extracellular matrix(ECM)**	Combined treatment with SPP1 inhibition and PD-1 inhibitors	HCC		[98]
	AZD4547(FGFR inhibitors)	Lungs adenocarcinoma		[96]
	Slit2(Activation of M1 macrophages)	Breast Cancer		[102]
**Extracellular Vesicle (EV)**	M1 macrophage-derived exosomal ncRNAs	Not specified		[179]
	Rab22a-NeoF1 fusion protein inhibitor (Blocking exosomes produced by tumor cells)	Pulmonary metastasis		[122]
	Use of FRβ (folate receptor beta)-specific CAR-T cells	Not specified		[132]
	Disrupts Treg cell-TAM axis and targets pathways such as SREBP1	Not specified		[145]
TAMs, tumor-associated macrophages; CRC, colorectal cancer; HCC, Hepatocellular carcinoma; EMT, epithelial -to-mesenchymal transition; ECM, Extracellular matrix; EV, extracellular vesicle; Treg cell, regulatory T cell; NCT, ClinicalTrials.gov.				

## Conclusions, and perspectives

In this review, we delve into the roles and distributions of TAMs within the TME, highlighting their critical functions in tumor progression and the exploration of their principal influencing factors. TAMs exhibit dynamic changes and a unique distribution that significantly impact cancer progression. The intratumoral heterogeneity of the TIME and the spatial arrangement of immune cells are increasingly recognized as correlating with disease progression and clinical-pathological factors [180]. TAMs dynamically regulate their functional expressions and interact with tumor cells, immune cells, and stromal cells, thereby promoting tumor progression. Advances in single-cell sequencing and multi-omics technologies have enabled us to delineate various subtypes and functions of TAMs, enhancing our understanding of their clinical relevance.

The diversity in subtypes and distributions of TAMs is linked to patient prognoses and associated with tumor treatment responses and resistance. By reviewing and analyzing the status of TAMs across different cancer types, integrating this data aids in understanding TAM functions, selecting suitable therapeutic targets, and devising precise intervention strategies. This review also outlines the current methodologies for studying the spatiotemporal heterogeneity of TAMs, presenting these in Table 1 for reference.

Leveraging findings from these studies can deepen our comprehension of cancer development and guide the formulation of effective therapeutic strategies. Research indicates that exploiting the biological properties of TAMs can significantly improve disease control across various cancer types [20]. Presently, the primary strategy for targeting macrophages involves the CSF-1/CSF-1R or CCL2/CCR2 pathways, though their effects remain somewhat limited [3, 181]. It is imperative to develop more potent targeting approaches. Recent studies have shown promising results using TREM2-specific antibodies in conjunction with commonly used anti-PD-1 antibodies, highlighting potential new avenues for targeted therapy [176]. This review also discusses potential targeted therapeutic strategies against TAMs that may enhance the development of novel drugs, boost immunotherapy efficacy, and improve patient outcomes. Understanding the dynamics of TAMs within the TME is crucial for crafting effective treatment protocols. We aim to apply our understanding of TAMs’ spatiotemporal heterogeneity in clinical decision-making and explore effective treatments based on TAM biology to benefit patients.

## Data Availability

No datasets were generated or analysed during the current study.

## Abbreviations

TAMs: Tumor-associated macrophages
TME: Tumor microenvironment
TRMs: Tissue-resident macrophages
MDMs: Monocyte-derived macrophages
EMT: Epithelial-mesenchymal transition
CAFs: Cancer-associated fibroblasts
EVs: Extracellular vesicles;
ECM: Extracellular matrix
ECs: Endothelial cells
mIHC: Multiplex immunohistochemistry
CSC: Cancer stem cells
CRC: Colorectal Cancer
HCC: Hepatocellular carcinoma
TLS: Tertiary lymphoid structures
scRNA-seq: Single-cell RNA sequencing
DCs: Dendritic cells
